# Pulmonary Function and Hospital Admission in Patients with Cystic Fibrosis Based on Household Second-Hand Smoking

**Published:** 2018-01

**Authors:** Maryam Hassanzad, Shabnam Eslampanah, Mohammadreza Modaresi, Sabereh Tashayoie-Nejad, Ali Akbar Velayati

**Affiliations:** 1 Pediatric Respiratory Diseases Research Center, National Research Institute of Tuberculosis and Lung Diseases (NRITLD), Shahid Beheshti University of Medical Sciences, Tehran, Iran.; 2 Tobacco Prevention and Control Research Center, National Research Institute of Tuberculosis and Lung Diseases (NRITLD), Shahid Beheshti University of Medical Sciences, Tehran, Iran,; 3 Pediatric Pulmonary Disease and Sleep Medicine Research Center, Pediatric Center of Excellence, Children’s Medical Center, Tehran, Iran.

**Keywords:** Pulmonary function test, Cystic fibrosis, Second-hand smoking

## Abstract

**Background:**

Smoking is a known predisposing factor to exacerbations in CF patients. But the effects of second-hand tobacco smoking are not yet clear. Hence, this study determined the clinical and spirometric presentations and urinary cotinine levels among cystic fibrosis patients over seven years of age in relation to their parent’s smoking history.

**Materials and Methods:**

In this cross-sectional comparative study, 58 consecutive cystic fibrosis patients older than seven years of age were enrolled. These patients were divided into two equal groups: those with second-hand tobacco smoking and those without. Pulmonary function tests and hospital admission rates were compared across the groups.

**Results:**

The mean hospital admission times were 5.1±2.4 in the group with passive smoking, and 2.6±1.3 times in the group without (P<0.001). The cotinine level was reversely correlated to time interval passed from previous admissions (P=0.001, r=−0.432) in passive smokers and (P=0.021, r=0.314) in non-passive smokers. In the analysis of FEV1 with urine, there was a significant but negative relation between FEV1 and cotinine (P= 0.002). Besides, in the analysis of FE 25–75 and urine cotinine, there was also a significant and negative relationship (P=0.001).

**Conclusion:**

From our findings, we conclude that pulmonary function tests and hospital admission rate in patients with cystic fibrosis are associated with urinary cotinine level and household second-hand tobacco smoking.

## INTRODUCTION

Cystic fibrosis (CF) is the most common hereditary respiratory disease of childhood with autosomal–dominant pattern of transition and increasing trend of incidence (1–3). The subjects with CF and their caregivers and families usually have a decreased quality of life (4–7). Also, high economic costs and other socio-cultural conflicts may be seen in families with affected members (8–11).

The available therapeutic approaches are generally conservative and complete cure for these patients is not accessible (12–15). Accordingly, preparing optimal situations to develop better life status in patients with CF is essential (16, 17). Determination of the factors contributing to both mental and somatic health status is important (18). Smoking is a known predisposing factor for exacerbations in CF patients (19, 20), but the effects of second-hand smoking are not yet clear. Hence, this study determined the clinical and spirometric presentations and urinary cotinine levels among cystic fibrosis patients over seven years of age according to their parent’s smoking history.

## MATERIALS AND METHODS

In this cross-sectional comparative study, 58 consecutive cystic fibrosis patients older than seven years of age were enrolled. These patients were divided into two equal groups: those with second-hand smoking and those without.

The age, sex, body mass index (BMI), family history of respiratory diseases, symptoms in patients, chief complaints, computed-tomography (CT) scan findings, clubbing severity, spirometric parameters, the times passed from last admission, relative status of parents, smoking by parents and cigarette packs they used per year (according to IUATLD Questionnaire) were assessed in the two groups. Two main variables — annual hospital admission times and pulmonary function test — were evaluated and compared across the groups. Also the association of different factors was compared with these two variables.

Data analysis was performed among 58 subjects: 29 patients with second-hand smoking and 29 patients without. Cotinine level was evaluated with ELISA test. Data were analyzed using SPSS (version 20.0) software (Statistical Procedures for Social Sciences; Chicago, Illinois, USA). Chi-square, Kendall, and independent-sample *t*-tests were used; P values less than 0.05 were considered statistically significant.

## RESULTS

The age, sex, BMI, family history of pulmonary disease, and relative status of parents were the same across the groups (Table 1). Only fathers were smokers, smoking 5.9±9.9 packs per year. The symptoms, CT scan findings were similar across the groups (P>0.05) (Tables 2 and 3).

**Table 1.** Demographic characteristics in two groups

**Variables**	**Total**	**No Passive Smoking N=29**	**Passive Smoking N=29**	**P-Value**
**Age**	15.77±6.93	16.86±7.29	14.67±6.49	0.232
**Sex**				
Male	36(62.1%)	17 (58.6%)	19 (65.5%)	0.588
Female	22(37.9%)	12 (41.4%)	10 (34.5%)	
**BMI**				
Mean±SD	16.65±4.36	15.68±3.08	17.63±5.21	0.155
<18	41 (70.7%)	23(79.3%)	18 (62.1%)	
18–24	15(25.9%)	6(20.7%)	9 (31.0%)	0.216
>24	2 (3.4%)	0(0%)	2 (6.9%)	
Normal	15 (25.9%)	6(20.7%)	9 (31.0%)	0.368
**Family history of Pulmonary Disease**	12 (20.7%)	6(20.7%)	6 (20.7%)	NS(>0.999)
**Relative Status of Parents Smoking**	34 (58.6%)	14(48.3%)	20(69.0%)	0.110
Mother	0	0	0	-
Father	29 (50.0%)	0	29(100.0%)	-
**Pack/Year**				
Mother	-	0	-	-
Father	5.89±9.90	0	11.77±11.46	-

**Table 2.** Clinical symptoms in two groups

	**Passive smoking**	**No Passive Smoking**	**P-Value**
Cough	27 (93.1%)	29 (100%)	0.1
Productive cough	21 (72.4%)	25 (86.2%)	0.1
Cyanosis	2 (6.9%)	-	0.1
Diarrhea	1 (3.4%)	1 (3.4%)	1.0
Abdominal Pain	2 (6.9%)	1 (3.4%)	0.5
Clubbing	28 (96.6%)	28 (96.6%)	1.0
Polyp in nose/sinus	17 (85.6%)	22 (75.9%)	0.1

**Table 3.** CT scan findings in two groups

	**Passive smoking**	**No Passive Smoking**	**P-Value**
Infiltration	7 (25%)	5 (17.2%)	0.4
Collapse	7 (24.1%)	2 (6.9%)	0.07
Pleural Effusion	1(3.4%)	-	0.3
Emphysema	11 (38.9%)	12 (41.4%)	0.7
Consolidation	4 (13.8%)	3 (10.3%)	0.6
Bornchiectasis	27 (03.1%)	25 (86.2%)	0.3

**Figure 1.**
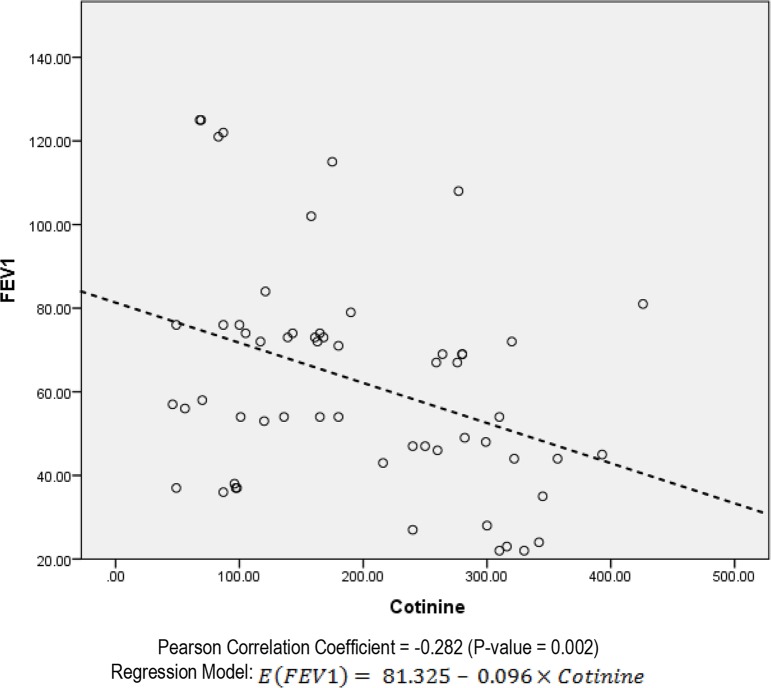
Scatter plot of the relation between FEV1 and urine cotinine in the patients with CF who are passive smoker

**Figure 2.**
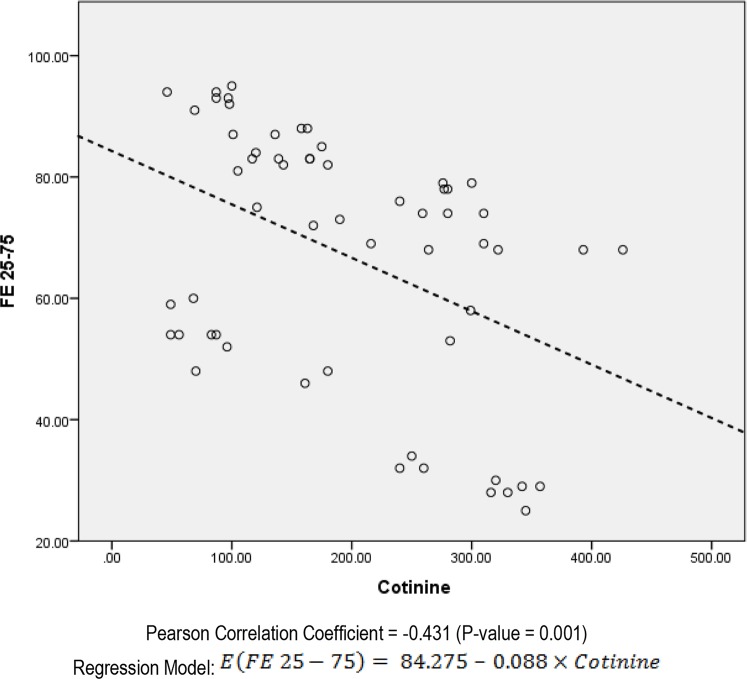
Scatter plot of the relation between FE 25–75 and urine cotinine in the patients with CF who are passive smoker

The mean hospital admission times were 5.1±2.4 times in group with passive smoking and 2.6±1.3 in group without (P<0.001). The cotinine level was reversely correlated to time interval passed from previous admission (P=0.001, r=−0.432) in passive smokers; and (P=0.021, r=−0.314) in non-passive smokers. In the analysis of FEV1 with urine, there was a significant but negative relation between FEV1 and cotinine (P=0.002) (Figure 1). In the analysis of FE 25–75 and urine cotinine, there was also a significant and negative relationship (P=0.001) (Figure 2).

## DISCUSSION

Patients with CF may show more respiratory response to air pollution in and out of home and in other closed environments. Smoking is a cause for exacerbation of disease leading to decreased quality of life in patients with CF (19, 20). However, the role of second-hand smoking is not yet clear. This matter is especially doubtful in spirometric and laboratory indices in CF subjects. In our study, urine cotinine level and hospital admission times were related to passive smoking. Also, the pulmonary function tests were correlated to amount of second-hand smoking. Interestingly, higher cotinine level was accompanied by shorter time intervals between hospital admissions. The applicability and usefulness of urinary cotinine is shown for monitoring pregnancy and other groups at risk, evaluating the impact of smoking cessation programs, validating phase I clinical trials, assessing occupational exposure to industrial pollutants, and the initial assessment of life insurance candidates (21).

In the study by Raju et al. (22), it was reported that Acrolein present in cigarette smoke could cause systemic dysfunction of cystic fibrosis transmembrane conductance regulator in extrapulmonary tissues. Rasmussen et al. (23) reported that cigarette smoke-induced Ca2+ release is the main cause of this systemic dysfunction.

The study by Campbell et al. (24) demonstrated that second-hand smoking was significantly associated with poorer spirometric results and a five-fold increase in the rate of hospitalization during the previous year. Their findings are similar to our study: the increase in the hospitalization rate in our report was two-fold in the control group. Kovesi et al. (25) reported that patients without passive smoking had higher spirometric measurements than subjects with second-hand smoking but, contrary to our findings, the difference was not statistically significant.

Smyth et al. (26) reported that passive smoking in cystic fibrosis patients was accompanied by impaired lung function tests. But contrary to our findings, they found that in the cystic fibrosis group urine cotinine was significantly lower than in the control subjects. Ortega-Garcia et al. (27) found that active smoking by pregnant mothers was associated with significantly lower spirometric measurements in young CF patients.

From our findings, it may be concluded that pulmonary function tests and hospital admission rates in patients with cystic fibrosis are associated with urinary cotinine levels and household second-hand smoking. However, further multi-center studies with larger sample sizes are required to attain more definitive results. Also from our findings, it is recommended that tobacco cessation and prevention programs be integrated as an important component for parents with CF children.
